# Gender Differences in Skeletal Muscle Substrate Metabolism – Molecular Mechanisms and Insulin Sensitivity

**DOI:** 10.3389/fendo.2014.00195

**Published:** 2014-11-13

**Authors:** Anne-Marie Lundsgaard, Bente Kiens

**Affiliations:** ^1^Section of Molecular Physiology, Department of Nutrition, Exercise and Sports, August Krogh Centre, University of Copenhagen, Copenhagen, Denmark

**Keywords:** substrate metabolism, glucose uptake, fatty acid oxidation, intramyocellular triacylglycerol, exercise, metabolic flexibility, adipose tissue

## Abstract

It has become increasingly apparent that substrate metabolism is subject to gender-specific regulation, and the aim of this review is to outline the available evidence of molecular gender differences in glucose and lipid metabolism of skeletal muscle. Female sex has been suggested to have a favorable effect on glucose homeostasis, and the available evidence from hyperinsulinemic–euglycemic clamp studies is summarized to delineate whether there is a gender difference in whole-body insulin sensitivity and in particular insulin-stimulated glucose uptake of skeletal muscle. Whether an eventual higher insulin sensitivity of female skeletal muscle can be related to gender-specific regulation of molecular metabolism will be topic for discussion. Gender differences in muscle fiber type distribution and substrate availability to and in skeletal muscle are highly relevant for substrate metabolism in men and women. In particular, the molecular machinery for glucose and fatty acid oxidative and storage capacities in skeletal muscle and its implications for substrate utilization during metabolic situations of daily living are discussed, emphasizing their relevance for substrate choice in the fed and fasted state, and during periods of physical activity and recovery. Together, handling of carbohydrate and lipids and regulation of their utilization in skeletal muscle have implications for whole-body glucose homeostasis in men and women. 17-β estradiol is the most important female sex hormone, and the identification of estradiol receptors in skeletal muscle has opened for a role in regulation of substrate metabolism. Also, higher levels of circulating adipokines as adiponectin and leptin in women and their implications for muscle metabolism will be considered.

## Introduction

The number of diagnosed type 2 diabetic (T2D) patients is still increasing, and notably the global prevalence is reported to be higher in men than women (1). In biomedical research, it has become increasingly apparent that gender has a profound impact on metabolism, and it has been questioned whether female sex has a favorable effect on insulin sensitivity. Intriguingly, women present with around two-third the skeletal muscle mass and twice the adipose mass of their male counterparts, which would actually predispose for the opposite scenario. To answer the question, available evidence from well controlled human clinical trials will be analyzed, emphasizing studies where the hyperinsulinemic–euglycemic clamp (H–E clamp) has been applied to evaluate insulin sensitivity. Investigating the nature of primary biologic gender differences is difficult, as confounding variables as adiposity, fat distribution, hormonal status, and aerobic fitness level might complicate interpretations. Thus, matching of men and women in regard to body composition, maximal oxygen uptake (VO_2_-peak) per lean body mass (LBM), training status, and menstrual cyclicity is crucial in order to determine the effect of sex *per se*.

The majority of gender studies have evaluated insulin sensitivity at a whole-body level, which in turn reflects the combined sensitivity of liver, adipose tissue, and skeletal muscle. Skeletal muscle has been described as a quantitatively important site for insulin-stimulated glucose clearance, in studies evaluating peripheral glucose disposal by the leg arterio-venous (a-v) balance technique (2). This implicates the importance of investigating possible sex differences in insulin action in muscle. During a H–E clamp, the glucose infusion rate (GIR) can be expressed relative to the size of LBM, which gives a rough estimate of glucose clearance by skeletal muscle. However, to obtain valid conclusions, insulin sensitivity is to be measured in skeletal muscle. To that end, clinical trials conducted by our research group contribute with important information, measuring glucose uptake across the leg by applying the femoral a-v balance technique on carefully matched men and women.

In this review, we seek to explain whether a possible difference in skeletal muscle insulin sensitivity can be related to hormonal differences or gender-specific regulation of molecular metabolism in muscle. An increasing body of evidence suggests that there is a distinct gender dimorphism in the intrinsic properties of skeletal muscle. Notably, when gene expression was evaluated using large-scale microarray of human vastus lateralis muscle, gender was reported to have a stronger influence on gene expression than age and training status (3). Determining the causative factors as well as implications of gender differences in metabolic properties of skeletal muscle is highly relevant.

After puberty, the hormonal milieu is markedly changed, in turn mediating the distinct gender diversity in adiposity. 17-β estradiol is the most important female sex hormone, and it has become clear that its actions are wide. The identification of estradiol receptors in skeletal muscle has indeed opened for a role of this hormone in regulation of substrate metabolism. Also, a higher body fat deposition in women is likely to affect levels of circulating adipokines, which in turn may influence metabolism in skeletal muscle by receptor-binding.

Gender differences in muscle morphology, i.e., fiber type composition and capillarization, are likely to affect the capacity for oxidative versus glycolytic energy turnover. Furthermore, the availability of circulating as well as intramuscular substrates and the capacity for glucose and fatty acid (FA) uptake are relevant determinants of the relative utilization of FA and glucose. Proteins related to plasma membrane transport and the molecular machinery for glucose and FA storage and oxidation will be considered, emphasizing factors that impact on the relative utilization of glucose and FA in female and male skeletal muscle. Identifying possible players that regulate the preference between glucose and FA utilization, and whether these are subject to a gender-specific regulation is of key interest.

An overall definition of high insulin sensitivity in skeletal muscle is difficult to convey. It might not solely be a question of achieving a high rate of glucose uptake per muscle mass at a given time point. The actual substrate choice of the myocytes is highly dependent on cellular energy status and substrate availability. Thus, we aim to give a more nuanced picture of gender differences in the relative glucose and FA utilization in different situations, i.e., at rest, in the prandial state and during increased cellular energy turnover. In daily living, men and women will continuously fluctuate between the fed and fasted state, physical activity and rest. Thus, we think it is important to consider substrate choice in each of these situations, as this has an impact on overall glucose homeostasis.

## Whole Body and Skeletal Muscle Insulin Sensitivity

Several studies have measured whole-body insulin sensitivity or related markers in men and women, applying various methods. Fasting glucose levels in plasma reflect the interplay between basal whole-body glucose disposal and endogenous glucose production. In a large cross-sectional study including 1188 individuals, controlled for family history of T2D, the prevalence of impaired fasting glucose was reported to be higher in men than women (17 versus 13%) (4). When the impact of sex was further evaluated on insulin action in a cohort of ~8000 Swedish men and women by an oral glucose tolerance test (OGTT), the prevalence of impaired fasting glucose and diabetes was ~2-fold higher in men compared to women (5). Also, in a Danish population study of 380 individuals matched for age and body mass index (BMI), it was demonstrated that glucose clearance rate during an intravenous glucose tolerance test (IVGTT) was 15% higher in women compared to men (6). Thus, from these large cross-sectional studies, it appears that men might be more prone to develop insulin resistance.

In Table 1, clinical trials applying the H–E clamp in healthy subjects are summarized. In all studies, subjects were matched on age and BMI. Only studies including premenopausal women have been included. Some studies have also included VO_2_-peak in their matching criteria and considered menstrual cycle phase, and in some trials diet was controlled prior to the clamp. These variables may all influence metabolism and insulin sensitivity.

**Table 1:** **Summary of H–E clamp studies in healthy premenopausal women and men**.

Reference	Subjects	Matching	Menstrual status	Fitness level/VO_2_-peak	H–E clamp and dietary control	Insulin sensitivity
Yki-Jarvinen, 1984 (7)	13 Women, 21 ± 1 years old	BMI, age, VO_2_-peak/kg BW	Follicular	48 ± 1 and 52 ± 2 ml/kg/min	2 h	Women = men, when GIR expressed per kg BW
	11 Men, 23 ± 1 years old				40 mU/m^2^/min	
					2 days control diet	Women > men by 45%, when GIR expressed per kg LBM (*p* < 0.01)
Nuutila et al., 1995 (8)	7 Women, 29 ± 2 years old	BMI, age, VO_2_-peak/kg BW	Follicular	39 ± 4 and 44 ± 3 ml/kg/min	2 h	Women > men by 41%, when GIR expressed per kg BW (*p* < 0.05)
	9 Men, 31 ± 2 years old				1 mU/kg/min	
					3 days control diet +^18^FDG/PET scan	47% higher glucose uptake in muscle of women
Donahue et al., 1996 (9)	13 Women, 37 ± 5 years old	BMI, age	Not considered	Not considered	2 h	Women = men, when GIR expressed per kg BW
	15 Men, 33 ± 5 years old				40 mU/m^2^/min	
					No dietary control	Women > men by 46%, when GIR expressed per kg LBM (*p* < 0.05)
Sumner et al., 1999 (10)	24 Women	BMI, age	Follicular	Not considered	2 h	Women > men, when GIR expressed per kg LBM (*p* < 0.01)
	31 Men				40 mU/m^2^/min	
	Mean age 32 ± 4 years old				No dietary control	
Frias et al., 2001 (11)	8 Women, 42 ± 8 years old	BMI, age	Follicular	Not considered	5 h	Women = men, when glucose Rd expressed per kg BW
	10 Men, 35 ± 6 years old				80 mU/m^2^/min	
					No dietary control	
Perseghin et al., 2001 (12)	15 Women, 26 ± 1 years old	BMI, age	Follicular	Physical activity index 8.9 and 9.2 (3–15 scale)	1 mU/kg/min	Women = men, when GIR expressed per kg LBM
	15 Men, 24 ± 1 years old				2 h	
					3 Week isocaloric diet	
Rattarasarn et al., 2004 (13)	11 Women, 39 ± 9 years old	BMI, age	Not considered	Not considered	2 h	Women = men, when GIR expressed per kg BW
	11 Men, 41 ± 7 years old				50 mU/m^2^/min	
					No dietary control	
Borissova et al., 2005 (14)	21 Women	BMI, age	Not considered	Not considered	2 h	Women > men by 38%, when GIR expressed per kg BW (*p* < 0.001)
	23 Men				1 mU/kg/min	
	<40 years old				No dietary control	
Soeters et al., 2007 (15)	10 Women, mean age 21	BMI, age	Follicular	Sedentary	5 h	Women = men, when glucose Rd expressed per kg BW
	10 Men, mean age 22			<3 h/week	60 mU/m^2^/min	
					38 h fast preceded by 3 days isocaloric diet	
Shadid et al., 2007 (16)	35 Women, 39 ± 8 years, BMI 28 ± 7	BMI, age, VO_2_ -peak/kg LBM	Not considered	49 ± 10 and 52 ± 10 ml/kg LBM/min	1 mU/kg LBM/min	Women = men, when GIR expressed per kg BW
	28 Men, 33 ± 8 years, BMI 29 ± 6				5 days control diet	
Koska et al., 2008 (17)	21 Women	BMI, age	Follicular	No info	100 min	Women = men, when GIR expressed per kg estimated metabolic body size
	32 Men				40 mU/m^2^/min	
	Mean age 25				3 days control diet	
	BMI 36 ± 4	
Vistisen et al., 2008 (18)	8 Women	BMI, age, VO_2_-peak/kg LBM	Follicular (day 10)	44 ± 2 and 48 ± 1 ml/kg LBM/min	4 h	Women > men, when GIR expressed per kg LBM (*p* < 0.01)
	8 Men				40 mU/m^2^/min	
	42 ± 1 years old BMI 33 ± 1				3 days control diet	
Hoeg et al., 2009 (19)	8 Women, 24 ± 1 years old	BMI, age, VO_2_-peak/kg LBM	Follicular (day 7–11)	63 ± 2 and 63 ± 1 ml/kg LBM/min	2 h	Women > men by 22%, when GIR expressed per kg LBM (*p* < 0.05)
	8 Men, 25 ± 1 years old				1.1 mU/kg BW/min	
					8 days control diet	
						35% higher leg glucose uptake in women (*p* < 0.05)
Karakelides et al., 2010 (20)	12 Young lean	BMI, age, VO_2_-peak/kg LBM	Not considered	Young subjects: 46 and 47 ml/kg/min	8 h	Women > men, when GIR expressed per kg LBM (*p* < 0.05)
	12 Young obese				1.5 mU/kg LBM/min	
	12 Older lean			Older subjects: 30 and 31 ml/kg/min
					3 days control diet	
	12 Older obese	
	6 Women, 6 men in each group	
Hoeg et al., 2011 (21)	8 Women, 25 ± 1 years old	BMI, age, VO_2_-peak/kg LBM	Follicular (day 7–11)	62 ± 2 and 63 ± 1 ml/kg LBM/min	7 h	Women = men, when GIR expressed per kg LBM
	8 Men, 25 ± 1 years old				1.42 mU/kg BW/min	
					8 days control diet	29% higher leg glucose uptake in women (*p* < 0.05)

*Unless noted, subjects were normal weight (BMI 18–25 kg/m^2^). Data on age, BMI, and VO_2_-peak are expressed as mean ± SE. If possible, the relative difference in GIR between women and men is calculated*.

It appears that a significant part of the H–E clamp studies report a relative higher insulin sensitivity in women when GIR is expressed per kilogram LBM, although not a solely consistent finding. In a few studies, insulin sensitivity has been evaluated directly in skeletal muscle. When the forearm a-v balance technique was applied to healthy women after a 75 g oral glucose load, 3 h glucose uptake related to forearm muscle mass was ~37% higher in women compared to men (22). In a later study by Nuutila et al., a 47% greater rate of glucose uptake was observed in muscles of women compared to men, measured by positron emission tomography scanning during hyperinsulinemic conditions (8). We have conducted H–E clamp studies, applying the femoral a-v balance technique on carefully matched men and women, and found that women in the follicular phase had 29–35% higher insulin-stimulated glucose uptake in leg skeletal muscle (19, 21). Thus, it seems to be a consistent finding that there is a higher glucose uptake in female skeletal muscle when stimulated by physiologic insulin concentrations.

## Estrogen and Estrogen Receptors in Skeletal Muscle

Estrogens constitute a group of female sex steroid hormones together with progesterone. The endogenous forms of estrogens are 17β-estradiol, estrone, and estriol, of which 17β-estradiol is the most important. Importantly, the estrogen receptors α (ERα) and β (ERβ) are expressed in human skeletal muscle, as demonstrated at the gene expression level (23, 24). When immunohistochemistry was applied on human muscle biopsies it was demonstrated that ERα and ERβ proteins are localized to the myofiber nuclei and that their expressional level are independent of sex (25). ERα appear to be the prominent isoform, as mRNA of ERα is reported to be 180-fold higher than ERβ mRNA in vastus lateralis biopsies from human subjects (24). When human skeletal muscle cells were treated with estradiol for 24 h, mRNA of ERα, but not ERβ, was increased (26), and thus only ERα seems to be regulated by female sex hormones. Interestingly, both ERα and ERβ mRNA were reported to be 3–5-fold higher in endurance trained men compared to moderately active men, suggesting a role of ERs in the adaptations to exercise in skeletal muscle (27).

Estrogen receptors α and ERβ bind as homodimers at specific DNA motifs termed estrogen response elements. In addition, ERα can indirectly activate or repress transcription by binding to other DNA binding proteins. Moreover, estrogen actions may also be induced by non-genomic effects mediated by extranuclear ERs. Milanesi et al. has reported robust evidence for a mitochondrial location of ERα in the C2C12 mouse skeletal muscle cell line, demonstrating labeled estradiol binding to mitochondrial fractions by immunocytological staining (28), and hence a role for ERα in human mitochondria remains to be elucidated. A mitochondrial location of ERs might contribute to explain a higher gene expression of these in the endurance trained state, as observed in the study by Wiik et al. Interestingly, it has been demonstrated in rats that activation of ERα in skeletal muscle with 3 days of treatment with the selective ligand propylpyrazoletriyl increased insulin-stimulated glucose uptake in skeletal muscle (29). Together, findings underscore a role of estradiol and ERα for glucose metabolism in skeletal muscle.

## Role of Estradiol Levels

A growing body of evidence underscores the importance of estradiol in the regulation of metabolism. A study in adolescents has demonstrated that the gender difference in whole-body insulin sensitivity arises after puberty. A longitudinal study was conducted on a large cohort of adolescents, examined with repeated H–E clamps three times between the age of 11 and 19 years. Insulin sensitivity expressed per kilogram LBM showed a divergent pattern as it decreased in male adolescents during puberty, while it increased in female adolescents and became significantly higher in women than men at the age of 19 years (30). Interestingly, when plasma estradiol levels were acutely increased ~200% by an intravenous 2.5 mg estrogen bolus administered at baseline of a a H–E clamp, insulin action was increased by 20% in post-menopausal women when compared to a saline trial (31). These studies point to a role of estradiol in the regulation of whole-body insulin sensitivity *in vivo*, although the mechanisms are not clear. Later in life, when menopause causes the cessation in female sex hormone production, a gradual increase in susceptibility to metabolic complications and the metabolic syndrome is described (32). However, whether the incidence of insulin resistance or T2D increases in the menopausal transition will be beyond the scope of this review. An increased amount of evidence from longitudinal clamp studies following women in the pre-peri and post-menopausal state is required to make solid conclusions. Still, confounding variables as age and changes in physical activity have to be considered, and the role of estradiol *per se* may be impeded by changes in other sex hormones.

Throughout each menstrual cycle phase, the levels of female sex hormones undergo profound changes. Hence, plasma estradiol concentrations in women vary in the range from 10 to 300 pg/ml, peaking at the end of the follicular phase. Notably, circulating estradiol levels in men are indeed also of significance, considering the normal adult range of 40–50 pg/ml, and implying a possible role in male metabolism. It has been found in men that a mutation in the aromatase gene, which catalyzes the last step in the biosynthesis of estradiol from androgens, leads to a diabetic phenotype (33), and inborn mutation of the ERα gene in men is associated with insulin resistance (34). Thus, the relevance of estradiol for glucose homeostasis appears to extend to men. Though estradiol concentration in premenopausal women is subject to large during the menstrual cycle phase, no changes have been reported in whole-body metabolic rate and respiratory exchange ratio (RER) at rest (35), basal plasma glucose and insulin (36), or FA concentrations (37) when the follicular and luteal phase are compared. Also, when insulin sensitivity was assessed during the menstrual cycle in young, healthy women by an IVGTT, no differences in insulin sensitivity were observed between the luteal phase and the mid-follicular phase (38). Others have, however, reported a slight decrease in the luteal phase, as determined by homeostatic HOMA-IR (39), or IVGTT (40, 41), but it appears that whole-body insulin sensitivity in women are not subject to major changes during the menstrual cycle.

Today many women use oral contraceptives (OC) that modify hormonal status. The active estrogen is ethinyl estradiol, which is reported to the most potent of the estrogen agonists (42). OC use reduces natural estrogen production, and depending on OC type, three to five times more exogenous estrogen is provided compared with normal endogenous estrogen concentrations (43). In the 1960s, the ethinyl estradiol concentration in OCs were close to 150 μg, but were later decreased due to adverse effects as insulin resistance, and today 20–30 μg is the common dose. Still, the use of OCs might have implications in regard to glucose metabolism in women. In a cross-sectional study of 380 young healthy Caucasians, OC use was an important determinant of glucose effectiveness during an IVGTT, and notably as important as VO_2_-peak (6). Also, when a group of young healthy women using OC was compared to non-OC users matched for BMI, body composition and physical activity, and insulin sensitivity evaluated by a H–E clamp was reported to be 40% lower in the OC users (12). At physiologic levels, estradiol may positively influence whole-body insulin sensitivity, but excursion of estradiol concentrations outside its physiologic window may affect glucose metabolism and promote insulin resistance. In relation to this, when male to female transsexuals were treated with a large oral dose ethinyl estradiol for 4 months, glucose disposal during a H–E clamp decreased ~22% (44).

It could be speculated whether male sex steroids could be involved in the regulation of insulin sensitivity and substrate metabolism in both men and women. As for estradiol, excursion of testosterone and other androgens outside its normal range, appear to negatively influence glucose metabolism, as observed for women with the endocrine disorder polycystic ovarian syndrome (PCOS) (45). The effects of androgens on substrate metabolism should be considered in a separate review to extensively cover this topic.

## Adiposity and Adipokines

There is an obvious gender difference in adiposity, which is present already at birth (46) and becomes more marked during puberty (47). Varying with each decade, a 6–12% higher body fat was observed in women when a US cohort of 16,000 12–80 years old men and women was analyzed by bioelectrical impedance (48). Actually, the mean body fat percentage for normal-weight women is similar to men who are classified obese (49). It follows that a relatively lower LBM in women would in turn decrease capacity for systemic glucose clearance. Notably this is contrary to the sum of evidence from the mentioned H–E clamp studies, and it can be speculated why this is not so. It has been demonstrated with [18^F^]-FDG glucose and combined PET/CT scan that the absolute rate of insulin-stimulated glucose uptake in visceral and subcutaneous white adipose tissue is up to 40% of that in skeletal muscle (50). Thus, glucose uptake of the adipose tissue compartment does indeed contribute to systemic glucose clearance, and it seems that the role of the adipose compartment in glucose clearance has been underestimated. Interestingly, a higher basal as well as insulin-stimulated methyl-glucose uptake was observed *in vitro* in female versus male subcutaneous adipocytes, when expressed per cell number (51). These findings opens for a particular significant role of adipose tissue in whole-body glucose uptake in women, and it follows that gender differences in glucose uptake and metabolism of adipose tissue *in vivo* await further comparative studies. Importantly, body fat is also distributed differently in men and women, as described with the prevailing terms “apple” and “pear” shape. Men have a higher amount of visceral adipose tissue, whereas women have more peripheral subcutaneous fat, measured by CT scanning (52, 53). Visceral and subcutaneous fat differ markedly in histology and metabolism. The secretion of hormones and cytokines may be depot-dependent, and in particular the visceral depot has a higher rate of catecholamine-induced lipolysis and drainage of metabolites is directly subjected to the liver via the portal vein, which over time might have implications for hepatic lipid content and in turn hepatic insulin sensitivity (54). It can be hypothesized that the preferential subcutaneous fat distribution in women attenuates the propensity for hepatic lipotoxicity, which in turn protects hepatic glucose regulation.

The greater adiposity in women is likely to affect adipokine production and secretion. Serum leptin concentrations have been reported to be up to four times higher in premenopausal women than men (55). The gender difference in leptin levels becomes more marked after puberty (56), and is reported to persist after controlling for total body fat (57) and the relative amount of visceral or subcutaneous adipose tissue (58). Whether leptin plays a role in muscle metabolism warrants further investigation, but leptin receptors (OB-Rb) have been identified in human skeletal muscle. When plasma leptin was increased fourfold in female rats by 2 weeks of leptin infusion, a 2.5-fold increase in fat oxidation during contractions was observed in soleus muscle (59). Later, the increased fat oxidation was explained by a leptin mediated increase in phosphorylation of 5′AMP activated protein kinase (AMPKα2) and its downstream target acetyl-coA carboxylase (ACC) (60). The markedly higher circulating leptin concentrations in women are likely to have significant implications, and could be speculated to play a role in the regulation of fat oxidation in female skeletal muscle. In particular, it would be relevant to evaluate possible gender differences in OB-Rb expression and leptin signaling.

Another important adipokine is adiponectin, of which plasma concentrations are positively associated with whole-body insulin sensitivity (61, 62). We have observed 127% higher serum total adiponectin concentration in lean young women compared with matched men (63). This gender difference appears to be a consistent finding. Two cross-sectional studies including 1023 and 967 subjects have shown that median serum adiponectin levels were 56 and 88% higher in women than men (64, 65) and in a third study including 182 subjects, plasma adiponectin levels were reported to be 37% higher in women than men (66). Finally, a gender difference in plasma adiponectin concentration of 34 and 71% was reported in obese and non-obese subjects, respectively (67). In these four studies, the positive correlation between circulating adiponectin and whole-body insulin sensitivity was confirmed, and support a role for adiponectin for enhanced insulin sensitivity in women. Similar to leptin, the question is whether the higher adiponectin levels in women have direct implications for skeletal muscle metabolism. It has been demonstrated in C212 myocytes that adiponectin stimulates AMPK activity (68), and adiponectin induced AMPK activation was shown to increase glucose uptake and fat oxidation via inhibition of ACC in rat skeletal muscle (69). It could be speculated if the higher adiponectin concentrations in women could increase glucose uptake in skeletal muscle via AMPK dependent mechanisms. We have been able to demonstrate a correlation between serum adiponectin concentration, leg glucose uptake, and AMPK phosphorylation in men, but not in women (63), The lack of coherence in women could be related to their lower expression of adiponectin receptor 1 in skeletal muscle when compared to men, limiting the effects of the high serum adiponectin levels in women. Further studies are required to elucidate whether the gender diversity in adiponectin has direct metabolic effects in skeletal muscle that in turn could contribute to enhance glucose uptake.

## Muscle Morphology

A gender difference in muscle morphology has been well documented. By use of histochemical myosin adenosine triphosphatase (ATP-ase) staining we have observed a higher number of type I muscle fibers in the vastus lateralis muscle in women compared to matched men, and when expressed relative to area the proportion of type I fibers were 27–35% greater in women, while the proportion of type IIA (19, 70), or both IIA and IIX were reported to be greater in men (71). Hence, a greater muscle area is covered by type I fibers in women, in moderately (19, 72), as well as untrained and endurance trained matched men and women (71). We have also observed a larger individual fiber area of type IIA (70), or IIA and IIX fibers (19) in men. Others have confirmed a greater size of type II fibers in men compared to women and a greater ratio of type II to I fibers in men, also using myofibrillar ATP-ase staining (73–76). The immunohistochemical findings are reflected at the transcriptional level of the myosin heavy chains (MHC), as MHCI mRNA are reported lower in the vastus lateralis muscle of men than women (77), while MHCIIA and -IIX mRNA are higher in men (78). The number of capillaries surrounding each muscle fiber are found to be similar in men and women, but due to a lower total amount of type II fibers and a smaller individual area of these, a greater capillary density per given muscle area is observed in women (19, 70). Glucose and FA metabolism are highly dependent on enzymatic characteristics of the given muscle fiber. A greater capillary supply, and a greater area percentage of type I fibers in women are likely to enhance nutritive flow and increase oxidative glucose and FA metabolism, thereby contributing to gender differences in skeletal muscle metabolism and insulin sensitivity. An association between insulin sensitivity and the amount of oxidative type I fibers has been suggested, as a lower expression of type I fibers has been demonstrated in the vastus lateralis muscle of insulin resistant and T2D subjects as compared to healthy subjects (79, 80). Finally, both the amount of type I fibers as well as capillary density were well correlated to insulin action during a H–E clamp in lean and obese non-diabetic men (81).

## Availability of Circulating Lipid Substrates to Skeletal Muscle

### Plasma triacylglycerol and lipoprotein lipase

A gender difference in lipoprotein metabolism seems to be well established, as postprandial plasma triacylglycerol (TG) and very-low density lipoprotein-TG (VLDL-TG) concentrations are consistently reported to be lower in healthy lean and obese women than in men (82–85). It has been suggested that the lower postprandial concentrations in women are due to a higher clearance from plasma, as the rate of VLDL-TG secretion in the fasted state is actually reported to be higher (84, 86) or similar (87) in lean women compared to men, studied by intravenous infusion of glycerol and palmitate tracers or labeled 1-^14^C triolein VLDL-TG. The gender difference in TG clearance rate was actually suggested already in 1974, where Olefsky et al. showed that at a given VLDL-TG production, a lower plasma TG concentration was reported in women compared to men, measured in 53 subjects with a wide range of plasma TG concentrations (88). Thus, it appears that women extract more TG from plasma. Plasma TG is mainly cleared into adipose tissue, skeletal muscle, and heart. When TG clearance across the leg was investigated after a test meal with 34 E% dietary fat by use of a ^14^C-oleate tracer, TG extraction from plasma was demonstrated to be ~7- and ~3-fold higher in women than men in the overnight-fasted and fed state, respectively, and 6 h after the meal a higher ^14^C content was found in m. vastus lateralis of women compared to men (89). As the amount of TG cleared by subcutaneous adipose tissue was similar in men and women, the latter study indicates that the higher capacity for TG clearance is specific to female skeletal muscle. It appears that women may be more primed for lipid uptake into skeletal muscle, which in turn may influence plasma lipidemia after meals. In the prandial state, lower plasma TG excursions are indeed observed in women compared to men, investigated following ingestion of standardized high fat meals with 64 and 34 E% fat (83, 89) or evaluated during a whole day with several meals with 34 E% fat (90).

A greater clearance of TG from VLDL or chylomicron lipoproteins could be due to enhanced hydrolysis in the capillary bed of skeletal muscle, and thus muscle lipoprotein lipase (*m*LPL) may be an important player. The concentration of total LPL protein in plasma samples obtained in the fasted state is reported to be 35% higher in lean and obese healthy women compared to men (85). Furthermore, when fasting post-heparin total LPL activity was evaluated in plasma from ~500 men and women aged 17–64 years, a 30% higher LPL activity was reported in women (91). In skeletal muscle homogenates, we have reported 160% higher mRNA of *m*LPL in young women compared to matched males (92), and in support of this a 160% higher *m*LPL mRNA was also reported in skeletal muscle from 40 to 65 years old obese women compared to men (93). Whether the gender difference for the gene translates into a difference in *m*LPL protein expression is currently not known, as the lack of a specific antibody hinder further analyses of protein expression in men and women. When *m*LPL activity is evaluated in the overnight-fasted state, we have not been able to identify a gender difference in activity of *m*LPL (92). However, activity of LPL may differ in men and women when studied in other situations. Insulin increases adipose LPL (*a*LPL) activity (94), while it decreases *m*LPL activity (95), and it can be hypothesized that insulin-mediated suppression of *m*LPL is less in women in the fed state, due to their higher clearance of TG into skeletal muscle after meals. Hence, it would be of particular interest to gain more insight into the regulation of *m*LPL activity in the prandial state in women and men.

We have not evaluated *a*LPL activity, but others have demonstrated that women have a higher *a*LPL activity in both the fasted and fed state compared to men (96). A negative correlation has been reported between fasting *a*LPL activity and plasma estradiol concentration in healthy obese women (97, 98), suggesting that estradiol is a negative regulator of *a*LPL and hence TG clearance into adipose tissue. The regulation by estradiol has been investigated by treating premenopausal women in the early follicular phase with transdermal 17β-estradiol, using patches in the gluteal region, and it was found that *a*LPL protein expression and activity decreased in subcutaneous adipose tissue below the estradiol patches compared to placebo (99). Also, when adipocytes isolated from subcutaneous abdominal adipose tissue of healthy premenopausal women, were treated with 10^−7^ mol/L 17β-estradiol for 48 h, LPL protein expression was reduced (100). It remains to be investigated whether estradiol has inverse effects on LPL expression in female skeletal muscle, and thereby increases the potential for muscle lipid storage in women. Evidence to support this hypothesis can be derived from a study in rats, where it was found that acute estradiol infusion increased *m*LPL activity in the red vastus muscle, while reducing LPL activity in adipose tissue (101). Taken together data indicate that women have a higher clearance of plasma TG, and the generated FAs from the hydrolysis of the TG-rich lipoproteins by LPL seems to be taken up particularly into skeletal muscle. Whether this can be linked to a higher prandial *m*LPL activity in women than in men remains to be clarified.

### Plasma fatty acids

The concentration of plasma FA may also be a determinant of lipid availability for skeletal muscle. The available evidence suggests that adipose tissue in women is more sensitive to lipolytic stimuli (102), when subjected to metabolic stress as fasting or exercise (103) compared with men. Some, but not all, studies report a higher postprandial plasma FA concentration in women than men (104, 105), but it is possible that the divergence in findings can be related to lack of dietary control or improper matching of subjects. In a large systematic review, including 43 studies conducted after 1990, which have reported overnight-fasted plasma FA concentrations, BMI, and sex in healthy lean and obese subjects (953 women and 1410 men), it was found that plasma FA concentration was significantly higher in women (median 517 versus 434 μmol/L in men) (106). When healthy men and women were fasted for 48 h, plasma FA concentration increased 30% more in women than in men (107), and when the length of fasting was increased to 72 h, serum FA was reported to be 81% higher in women compared to men (108). Thus, it appears that the gender difference in circulating FA levels becomes more pronounced with prolonged fasting. When FA turnover is further investigated by applying 2.2-^2^H_2_-palmitate or U-^13^C-palmitate tracers, the gender difference is also confirmed. A 35–55% higher FA rate of appearance was observed in women compared to men, when evaluated for 12 h the day following an overnight fast (84). Similarly, when lean and obese men and women were followed in the isocaloric state for four consecutive days, the postabsorptive FA release, related to resting energy expenditure, was ~40% higher in women (109). Finally, it was demonstrated in 106 healthy men and women, with BMIs in the range of 18–44, that FA rate of appearance related to LBM was higher in women than men (110). It was demonstrated that FA release into plasma was similar in men and women when expressed per unit of fat mass. Together the findings demonstrate that women have higher postprandial FA concentrations, in particular in the fasted state, and thereby a higher FA availability per unit of their LBM, as a result of their higher fat mass than men.

### Fatty acid uptake across plasma membrane

A higher availability of FA to skeletal muscle may enhance lipid storage when capacity for FA uptake into this tissue is concomitantly increased. Skeletal muscle FA uptake occurs via passive diffusion but is also being mediated by lipid binding transport proteins (111), facilitating FA transport across the lipid bilayer. FAT/CD36 is the protein, which has been subject for most research, but also FATP1, FATP4, and FABPpm are involved in plasma membrane transport. A higher gene as well as protein expression of FAT/CD36 has been reported in women compared to men, irrespective of training status (92). Gene expression of FABPpm (92) and FATP1 (112) are observed to be higher in lean women compared to lean men, and a higher FABP_c_ mRNA expression has been reported in women compared to men (113, 114). Thus, at the protein level only FAT/CD36 has been confirmed to be higher in women than men, and it remains to be elucidated whether protein expression of the other lipid binding proteins also display gender differences. It is indeed possible that higher FAT/CD36 protein in women increase their capacity for FA transport. It seems that localization of this protein is important, as it has been demonstrated with the giant sarcolemmal vesicle technique that plasma membrane associated FAT/CD36 are highly correlated with intramyocellular (IMTG) storage in human skeletal muscle (115). Whether the amount of sarcolemmal-bound FAT/CD36 is higher in women has not been investigated.

## Lipid Metabolism in Skeletal Muscle

### Intramyocellular triacylglycerol

An enhanced FA uptake into female skeletal muscle will increase intracellular fatty acyl-CoAs available for reesterification, depending on cellular energy demands. The Kiens group was the first to demonstrate that IMTG content is higher in lean women compared to men (71, 116), and this finding was later supported by themselves and others (19, 97, 117, 118). It is indeed possible that higher IMTG concentrations in skeletal muscle of women can be coupled to their higher plasma FA availability and higher amount of FAT/CD36 in skeletal muscle. Also, a higher amount of type I muscle fibers in women is a factor to consider, as IMTG content is reported to be 2.8-fold higher in type I fibers compared to type II fibers (119). Notably, IMTG concentrations are often negatively correlated to whole-body insulin sensitivity in men, with the exception of athletes (120). It can be questioned why this relationship is different in women and whether it can be coupled to metabolic features similar to the endurance trained state. By use of electron microscopy, it has been found that IMTG in women is localized in a higher number of smaller lipid droplets compared to men (118). This morphologic characteristic might increase accessibility of lipases and proteins associated to the lipid droplets. Interestingly, lipid droplets in women were found to be located closer to mitochondria after an exercise bout (97), a location which may increase susceptibility to oxidation. The phospholipid surface of lipid droplets is covered with a number of proteins involved in lipid metabolism and trafficking of the lipid droplets. It has been demonstrated in untrained 40 years old men and women, matched for BMI and VO_2_-peak/kg LBM, that skeletal muscle protein expression of perilipin 2, 3, 4, and 5 (also known as ADRP, TIP47, S3-12, and OXPAT, respectively) is 1.5- to 2-fold higher in women (121). Of these, perilipin 3 may be considered important for lipid droplet lipolysis (122), and perilipin 5 has been described to interact with lipolytic key proteins as ATGL and is activator CGI-58 (123). Furthermore, recent work also indicates that perilipin 5 mediates an interaction between lipid droplets and mitochondria (124). Taken together, smaller lipid droplets and increased expression of perilipins in women are likely to increase association with lipases and eventually mitochondria, thereby increasing lipolytic turnover of IMTG. Whether the higher expression of the perilipins is simply due to a higher content of lipid droplets in women remains to be elucidated. It might also be that IMTG concentration *per se* is not an important determinant of insulin sensitivity in skeletal muscle. Instead, accumulation of lipid metabolites, such as diacylglycerol or ceramides, has been suggested to play a role, and it could be speculated whether there are gender differences for these other lipid fractions. Studies in this area are scarce, but one study has reported no difference in diacylglycerol or ceramide content between healthy men and women, though subjects had a wide age- and BMI range (125). Further studies are required to exclude a differential influence of lipid metabolites and related lipotoxicity in men and women.

### Intramyocellular triacylglycerol turnover

Increased TG storage in skeletal muscle of women is likely the result of increased esterification of FAs, as *de novo* lipogenesis is limited in human skeletal muscle. The ratio between FA oxidation and storage has been investigated in the prandial state. After a standardized meal (27 E% fat), containing ^3^H-triolein, was given to healthy premenopausal women and men, 24 h ^3^H_2_O derived FA oxidation was observed to be greater in men, suggesting a greater storage of FA in women (126). This is supported by the finding that non-oxidative FA disposal was demonstrated to be higher in women than men, when FA turnover was evaluated by infusion of 9–10,^2^H-palmitate and indirect calorimetri applied (127). These two studies suggest that at a whole-body level a significant greater share of exogenous FAs are stored, rather than oxidized, in women. More interestingly, when high plasma FA concentrations were induced by a 48 h fast, it was shown by ^1^H-MRS that TG increased in muscle of women, while TG increased in the liver of men (107), suggesting that the greater storage are specific to female skeletal muscle. This is supported by the findings of increased expression of genes linked to fat storage in skeletal muscle of women, such as the transcription factor sterol regulatory element binding protein 1c (SREBP-1c) (113, 114) and mitochondrial glycerol-3-phosphate acyltransferase (mtGPAT) (114), of which the latter is important for synthesis of the glycerol backbone for TG.

Stearoyl CoA desaturase 1 is important for desaturation of lipids in skeletal muscle and suggested to play a role in TG synthesis (128). It could be speculated that women have a higher expression and/or activity of stearoyl CoA desaturase 1 (SCD1) in muscle compared to men. In a Swedish health survey of 554 men and 295 women, a significant higher index of Δ9 desaturase activity, i.e., 16:0/16:1n-7 and 18:0/18:1n-7 ratios, was observed in serum cholesteryl esters (129), indicative of increased SCD activity at a whole-body level. More specifically, SCD1 mRNA is observed to be higher in skeletal muscle of women than men (130), and this gender difference seem to extend to adipose tissue, when SCD1 gene expression is analyzed in gluteo-femoral and abdominal adipose tissue derived preadipocytes (131). Gender differences in SCD1 protein expression or activity in skeletal muscle awaits further investigation. Interestingly, when FA composition of skeletal muscle TG was separated by thin layer chromatography and analyzed by gas–liquid chromatography, a lower amount of saturated FAs was observed in lean and obese women compared to men (132), which indeed could suggest a higher desaturase activity specific to female muscle. It can be hypothesized that a lower saturation of TG increases affinity of the lipases (133), thereby contributing to increase lipolytic turnover. Furthermore, it is possible that a more unsaturated profile of muscle lipids in women decreases the risk of myocellular lipotoxicity, and thereby increase insulin sensitivity, but these questions remain to be further investigated.

It seems that women are more equipped for FA esterification than men and studies of lipid droplet morphology and associated proteins suggest a higher capacity for lipolysis as well. In this context, we have demonstrated a higher IMTG use during submaximal exercise in women than in men, irrespective of training status (70, 71, 116). Thus, during cellular energy stress it appears that hydrolytic activity against IMTG is higher in women than men. When total TG hydrolase activity was measured at rest in skeletal muscle homogenates obtained in the overnight-fasted state, a twofold higher activity was reported in women compared to men, with no differences in DAG hydrolase activity (125). It can be questioned whether these findings can be related to gender-specific regulation of lipases in skeletal muscle. We have observed a similar protein expression of ATGL and CGI-58 in matched men and women (unpublished data). In regard to HSL, we have reported higher protein content in muscle from moderately trained women compared to matched men, but were not able to couple an increased lipolytic activity in women with increased HSL phosphorylation (117). Considering the findings of Moro et al. (125), it seems relevant to further study the gender-specific regulation of ATGL activity, but also the interaction between lipases and lipid droplets in men and women at rest and during exercise.

## Glucose Transport and Insulin Signaling

Insulin-stimulated glucose uptake appears to be higher in skeletal muscle of women than men, a finding that implies the relevance of studying gender differences in muscle glucose uptake and metabolism. We have observed a similar protein expression of glucose transporter 4 (GLUT4) in skeletal muscle of men and women (19), despite reports of higher gene expression in women compared to men (114, 130). GLUT4 gene expression seems to be subject to regulation by estradiol, as incubation of human myotubes with estradiol for 24 h was found to increase mRNA of GLUT4 eightfold (26). Furthermore, GLUT4 protein is markedly reduced in skeletal muscle of ERα (−/−) knockout mice, and immunofluorescence demonstrated a marked reduction of GLUT4 at the plasma membrane (134). Although we have not been able to identify a higher total protein content of GLUT4 in women, it may be hypothesized that gender-specific regulation of GLUT4 translocation or activity contribute to increased insulin-stimulated glucose uptake in female muscle. We have not been able to demonstrate any gender differences in protein expression of the insulin receptor or proximal insulin signaling via Akt or AS160 (19), but it remains possible that downstream insulin signaling or translocation, docking and fusion dynamics of GLUT4 vesicles with the plasma membrane are differently regulated in women than men.

Hexokinase II is another key protein involved in glucose uptake, and here we have observed a 56% higher hexokinase II (HKII) protein expression in women compared to men (21), which agrees with the finding of 2.4-fold higher HKII mRNA in women (114). In mice overexpressing HKII protein, 2-deoxy-glucose transport into muscle was increased under hyperinsulinemic conditions (135), and it can be hypothesized that increased intracellular phosphorylation of glucose facilitates its uptake and thereby contributes to an increased capacity for glucose uptake in women. It should, however, be noted that maximal HKII activity has been evaluated in muscle homogenates from matched men and women, and was found to be similar (136). Though, whether this reflects enzyme activity *in vivo* at physiologic glucose and insulin concentrations remains to be elucidated.

Glucose uptake into skeletal muscle may also be influenced by glycogen stores and the capacity for storage of glucose into glycogen. Glycogen content is not reported to be different between men and women. We have observed similar muscle glycogen in the resting overnight-fasted state after a controlled diet, determined both by PAS staining and biochemically in muscle extracts (70), and this finding is confirmed by others in both trained and untrained individuals (137). Gene expression of glycogen synthase (GS) is reported to be higher in women than in men (130), while differences in protein expression have not been evaluated. When GS activity was measured in muscle homogenates from overnight-fasted individuals, we have not been able to identify a difference between matched men and women when activity was expressed as % of I-form or fractional velocity % (unpublished data). Thus, it seems that women and men are similar in their capacity for storage of glucose.

## Mitochondrial Metabolism

### Mitochondrial fatty acid transport and beta-oxidation

Before entering mitochondria, FA from plasma and IMTG lipolysis are activated to fatty acyl-CoAs by acyl-CoA synthase. To be oxidized FA have to be converted to their acylcarnitine form to cross the outer mitochondrial membrane, a reaction catalyzed by carnitine palmitoyltransferase 1 (CPT-1). In well trained men and women, there are no differences in the level of total muscle carnitine at rest (138), while gender differences in muscle free carnitine during exercise has not been investigated. CPT-1 mRNA is reported to be higher in women (114), a finding which is also confirmed in myotubes obtained from females compared to males (139). CPT-1 protein content and enzyme activity, measured in intact mitochondria isolated from vastus lateralis muscle biopsies, were however reported to be similar in both untrained and trained men and women (140, 141).

In the mitochondrial matrix, fatty acyl-CoA enters the β-oxidation pathway, with the acyl-CoA dehydrogenases performing the first reaction. Long-chain acyl-CoA dehydrogenase (LCAD) mRNA is reported to be higher in women (114), while only very long- and medium chain acyl-CoA dehydrogenase (VLCAD and MCAD) protein expression is reported to be higher in women than men (142). Mitochondrial trifunctional protein (TFP) α catalyzes the second and third reaction for acyl-CoA substrates, while TFPβ catalyzes the fourth reaction in the production of acetyl-coA. The gene and protein expression of TFPα is reported to be higher in women (113, 142), while TFPβ protein seems to be similar in men and women (77). The hydroxy acyl-CoA dehydrogenase enzyme (HAD) catalyzes the third reaction, which leads to the production of NADH. When acyl-coA substrates is added to muscle homogenates, and the production of NADH is measured, the maximal activity of HAD seems to be similar in men and women (76, 143). Notably, glycolytic capacity appears to be greater in men. A higher activity of glycogen phosphorylase, pyruvate kinase, phosphofructokinase (PFK), and lactate dehydrogenase (LDH) has been demonstrated in muscle homogenates of young untrained men compared with women (144). These findings are supported in a later study, demonstrating a higher PFK, LDH, and malate dehydrogenase activity in muscles of men compared to women (145). Hence, men might have a higher capacity for glycogenolysis and glycolytic flux when compared to women, and a higher ratio between HAD activity and glycolytic enzyme activity is reported in women (144), suggesting increased potential for beta-oxidation than glycolysis in female muscle. It is indeed possible that the difference in glycolytic capacity between men and women can be related to the higher amount of type II fibers in men.

### Tricarboxylic acid cycle flux and mitochondrial ATP production

Acetyl-CoA substrates fuels the tricarboxylic acid (TCA) cycle and can be derived from β-oxidation as well as glycolysis. It can be questioned whether women have a higher capacity for NADH generation through the TCA cycle and ATP production from oxidative phosphorylation. We have found that citrate synthase activity is similar in skeletal muscle from matched men and women (19, 143), and activity of other important TCA enzymes as cytochrome *c* oxidase and succinate-cytochrome *c* oxidoreductase is also reported to be similar in men and women (146). When maximal ATP production was measured on freshly isolated mitochondria from skeletal muscle, using the luciferase reaction with different substrates, a similar ATP production rate was observed in lean and obese sedentary men and women, expressed relative to mitochondrial protein (20). Also, when mitochondrial respiration was analyzed in an oxygraph on muscle bundles, and complex I-, II-, III-, and IV-dependent respiration measured individually and related to either muscle weight, mitochondrial protein or citrate synthase, no gender differences were observed (147).

Together, the capacity for acetyl-coA flux through TCA and production of ATP from oxidative phosphorylation appears to be similar in men and women, suggesting an equal capacity for energy generation from glucose and FA. An increased β-oxidative to glycolytic capacity in women, concomitant with increased cellular availability of FA, will increase the propensity for a higher relative FA utilization in women, when ATP demands increases.

PDH is a key enzyme in the regulation of the metabolic switch between glucose and FA oxidation. PDK4 phosphorylates and thereby inhibits PDH, in turn inhibiting the conversion of pyruvate to acetyl-CoA, leading to enhanced fat oxidation and diversion of glycolytic intermediates to alternative metabolic pathways. When myotubes, obtained from females and males, were incubated with 17-β estradiol, PDK4 mRNA was reported to increase in female myotubes (139). Furthermore, 17-β estradiol treatment to ovariectomized female rats induced a 23-fold increase in PDK4 gene expression in skeletal muscle (148). Interestingly, a role for estradiol in the transcriptional regulation of PDK4 has been further confirmed in a human study. When muscle biopsies were obtained from monozygotic post-menopausal twins, of which one sister was a current user of hormone replacement therapy (HRT) (mean use 7 years), while her twin sister never used HRT, microarray analyses revealed that HRT was associated with a significant increase in the PDK4 gene (149). We are not aware of studies that have investigated differences in protein expression and regulation of PDK4 and PDH in men and women. Such possible gender differences in PDH activity with feeding and exercise may contribute to explain differences in regulation of glucose and FA oxidative flux in men and women.

## Substrate Turnover in Different Metabolic States

When studying substrate metabolism in men and women it is imperative to study how gender affects glucose and fat metabolism during the different metabolic situations of daily living, fluctuating between the prandial and postabsorptive state and periods with physical activity and recovery. In the resting overnight-fasted state, we have not been able to measure any gender differences in whole-body RER by indirect calorimetri, and it seems that postabsorptive glucose and FA utilization are similar in men and women. However, when substrate oxidation was then evaluated in metabolic chambers for 24 h, with three meals provided during the day, the rate of fat oxidation was lower in young women compared to men (150). In another study, where resting RER was evaluated by indirect calorimetri after two meals separated by 5 h, fat oxidation was observed to be consistently lower in women during the 10 h (126). These studies indicate that women utilize more carbohydrate than fat when they are in the prandial and postprandial state, likely due to the larger insulin sensitivity in women than in men. Indeed, more studies are needed to further investigate the prandial glycemic response in men versus women, in order to make conclusions on their respective insulin sensitivity in the fed state. Interestingly, when female and male endurance athletes increase carbohydrate intake from 55 to 60 to 75 E% for 4 days, muscle glycogen concentration was increased in men only after the isocaloric dietary manipulation (151), suggesting that the additional carbohydrates has been directed for oxidation rather than storage in women.

On the contrary, during conditions of increased energy demands women utilize more fat than carbohydrate to cover energy needs. It has been well described that women rely more on fat oxidation than men at the same relative submaximal exercise intensity. When calorimetric data from 25 studies comparing substrate oxidation in men and women during endurance exercise (>60 min) are summarized, mean RER indicates a moderately, but significantly, greater relative fat oxidation in women compared to men (114). Also, it has been demonstrated with indirect calorimetri during incremental tests on treadmill and cycle ergometer, that the rate of maximal fat oxidation is significant lower in men compared to women (152, 153). During submaximal exercise, a higher AMP/ATP ratio and greater AMPKα2 activation has been reported in moderately trained men compared to women (70), suggesting a better maintenance of myocellular energy balance in women during exercise, possible related to the more oxidative fiber type expression and better capillarization in women than in men. After exercise the opposite scenario appears to be in play. A recent meta-analysis, including 18 studies investigating substrate utilization in young men and women during 2–22 h of recovery from 60 to 120 min endurance exercise at 28–75% of VO_2_-peak, has reported a greater exercise-induced increase in lipid oxidation in men than women in the postabsorptive state (154). In support of this, we have observed a higher rate of glucose oxidation in women than men for 22 h after 120 min exercise at 55% VO_2_-peak (unpublished data). Furthermore, when FA tracers and indirect calorimetri were applied during 3 h of recovery from moderate intensity exercise at 45 or 65% of VO_2_max, fat oxidation was reported lower in women compared to men (155). Furthermore, when whole-body insulin action was assessed during the initial 3 h of recovery from 90 min exercise at 85% of lactate threshold by a H–E clamp, GIR was 27% lower in men compared to women (156). Together, these studies indicate that in the period following physical activity or exercise, FAs may be preserved for TG re-storage in women, while glucose is used to a greater extent to cover oxidative needs.

Taken together, when compared to men, women seem to utilize more carbohydrates in the fed state, as well as during recovery from physical activity, but less carbohydrate than men during exercise. As the fed state predominates in daily living, and the effects of physical activity might be prolonged, these observations suggest that insulin sensitivity and carbohydrate oxidation will be higher in women during the time course of a typical day. Differences in exercise substrate metabolism between males and females are likely influenced by sex hormones as discussed earlier and therefore one would expect differences between the two sexes to be less before puberty as well as after menopause. In agreement with this notion, differences in exercise substrate metabolism between women and men are not observed in childhood, but become evident with puberty (157). In addition, fat oxidation of post-menopausal women was 33% lower during exercise at 50% of VO_2_max compared to premenopausal women (158).

## Concluding Remarks and Perspectives

A significant part of the H–E clamp studies comparing men and women conclude that whole-body insulin sensitivity is higher in women, and it appears to be a solid finding that insulin-stimulated glucose uptake is higher in female skeletal muscle. Notably, this is observed despite greater body fat stores and greater lipid stores in skeletal muscle of women than in men. The molecular basis for the observation of higher insulin-stimulated glucose uptake in female skeletal muscle is not fully explained. An increased HKII-mediated glucose gradient, due to markedly higher HKII protein in women, may contribute to increase their capacity for glucose uptake, while it remains to be investigated whether GLUT4 translocation dynamics is subject to gender differences.

Gender differences in substrate metabolism have been well described. Women seem to oxidize more carbohydrates in the prandial state after meals, and when energy demands increases during physical activity, energy expenditure is covered by a greater fat oxidation in women. Together, these findings imply a high metabolic flexibility in women, as substrate oxidation is readily adjusted in accordance to nutrient availability in these situations. Both in the prandial phase and during recovery from exercise, FA substrates are directed toward TG storage, while glucose is directed for oxidation. At the molecular level, female skeletal muscle seems to be more “primed” for lipid storage as well as oxidation, which in turn contribute to keep the turnover of IMTG stores high.

The molecular differences in metabolism may reflect evolved adaptations in men and women that stem from differences in reproductive costs. Gestation and lactation is nutritionally expensive for women, and thus they would benefit from an increased ability to store fat in easily available depots as in skeletal muscle, thereby being more resistant to periods of food scarcity. In our days, where food plentiness prevails, the greater capacity for fat storage in women may result in better coping with lipid excess and thereby improved glucose tolerance. Interestingly, high plasma FA concentrations, obtained by intravenous infusion of Intralipid and heparin, induce significantly less (21) or no (11) reduction in whole-body insulin sensitivity in women compared to men, supporting an improved lipid handling in women. In the acute state, this might be coupled to more efficient direction of lipids toward storage in women, thereby decreasing cellular concentration of unesterified lipids, and the concomitant attenuation of glucose oxidation and metabolism in women.

More research is required to fully understand the role of gender in metabolism, and further unraveling of molecular mechanisms behind gender differences in glucose and lipid metabolism will contribute to enhance our understanding of acquired metabolic disorders as T2D. Future research areas include studies of glucose transport dynamics in skeletal muscle of men and women, and the causal mechanisms behind an increased metabolic flexibility in women. Also, gender differences in the relative contribution of adipose tissue to whole-body glucose disposal, remain to be investigated. In future metabolic research, it is worth considering the lack of convergence for gene and protein expression levels of many proteins and enzymes in female skeletal muscle. Notably, when gene trait analysis is applied to male and female skeletal muscle using microarray, a greater abundance of genes involved in RNA processing as ribosomal handling, transcription, and translation has been observed in women (159). This implicates that there might be a general higher turnover of mRNA in women than in men, and underscores the importance of studying metabolism at the protein level to make solid conclusions.

## Conflict of Interest Statement

The authors declare that the research was conducted in the absence of any commercial or financial relationships that could be construed as a potential conflict of interest.
